# SARS-CoV-2 Remdesivir Exposure Leads to Different Evolutionary Pathways That Converge in Moderate Levels of Drug Resistance

**DOI:** 10.3390/v17081055

**Published:** 2025-07-29

**Authors:** Carlota Fernandez-Antunez, Line A. Ryberg, Kuan Wang, Long V. Pham, Lotte S. Mikkelsen, Ulrik Fahnøe, Katrine T. Hartmann, Henrik E. Jensen, Kenn Holmbeck, Jens Bukh, Santseharay Ramirez

**Affiliations:** 1 Copenhagen Hepatitis C Program (CO-HEP), Department of Infectious Diseases, Copenhagen University Hospital, 2650 Hvidovre and Department of Immunology and Microbiology, Faculty of Health and Medical Sciences, University of Copenhagen, 2200 Copenhagen, Denmark; carlota.fernandez.antunez@regionh.dk (C.F.-A.); line.ryberg@sund.ku.dk (L.A.R.); kuan.wang@regionh.dk (K.W.); pham@sund.ku.dk (L.V.P.); lotte.scheibelein.mikkelsen@regionh.dk (L.S.M.); ulrik@sund.ku.dk (U.F.); kholmbeck@sund.ku.dk (K.H.); jbukh@sund.ku.dk (J.B.); 2 Department of Veterinary and Animal Sciences, University of Copenhagen, 1870 Frederiksberg, Denmark; katoh@sund.ku.dk (K.T.H.); elvang@sund.ku.dk (H.E.J.)

**Keywords:** SARS-CoV-2, broad-spectrum antiviral, DAA, nucleotide analog, remdesivir, resistance, fitness, mutation, Vero E6, hamster

## Abstract

Various SARS-CoV-2 remdesivir resistance-associated substitutions (RAS) have been reported, but a comprehensive comparison of their resistance levels is lacking. We identified novel RAS and performed head-to-head comparisons with known RAS in Vero E6 cells. A remdesivir escape polyclonal virus exhibited a 3.6-fold increase in remdesivir EC_50_ and mutations throughout the genome, including substitutions in nsp12 (E796D) and nsp14 (A255S). However, in reverse-genetics infectious assays, viruses harboring both these substitutions exhibited only a slight decrease in remdesivir susceptibility (1.3-fold increase in EC_50_). The nsp12-E796D substitution did not impair viral fitness (Vero E6 cells or Syrian hamsters) and was reported in a remdesivir-treated COVID-19 patient. In replication assays, a subgenomic replicon containing nsp12-E796D+nsp14-A255S led to a 16.1-fold increase in replication under remdesivir treatment. A comparison with known RAS showed that S759A, located in the active site of nsp12, conferred the highest remdesivir resistance (106.1-fold increase in replication). Nsp12-RAS V166A/L, V792I, E796D or C799F, all adjacent to the active site, caused intermediate resistance (2.0- to 11.5-fold), whereas N198S, D484Y, or E802D, located farther from the active site, showed no resistance (≤2.0-fold). In conclusion, our classification system, correlating replication under remdesivir treatment with RAS location in nsp12, shows that most nsp12-RAS cause moderate resistance.

## 1. Introduction

There has been an unprecedented effort to identify antivirals with activity against severe acute respiratory syndrome coronavirus 2 (SARS-CoV-2), due to the urgent need for therapies to mitigate the morbidity and mortality of the coronavirus disease 2019 (COVID-19) pandemic. The repurposing of existing antivirals led to the clinical investigation of the nucleotide analog remdesivir (also referred to as RDV or GS-5734) as a drug candidate for the treatment of SARS-CoV-2 infections [1]. Remdesivir is a prodrug of the adenosine analog GS-441524, which was discovered through the hepatitis C virus (HCV) pre-clinical antiviral programs [2]. Importantly, remdesivir is a broad-spectrum direct-acting antiviral (DAA) that targets the viral RNA-dependent RNA polymerase (RdRp). It has demonstrated antiviral efficacy against multiple viruses in cell culture and animal studies [3,4,5,6,7], including respiratory syncytial virus, Ebola virus, and coronaviruses such as SARS-CoV and MERS-CoV. Remdesivir exhibits high antiviral activity against SARS-CoV-2 in cell culture and pre-clinical animal models [8,9,10], whereas clinical trials and real-world data demonstrated that remdesivir treatment improved time to recovery and reduced mortality in COVID-19 hospitalized patients [11,12,13]. Remdesivir has been extensively used to treat COVID-19 patients requiring hospitalization in the United States and Europe since its approval in 2020, and it has also been approved for the treatment of non-hospitalized COVID-19 patients at high risk for progression to severe COVID-19 [14,15].

Considering the capacity of SARS-CoV-2 to evolve and acquire immune escape mutations that compromise vaccine efficacy [16], it is essential to understand the evolution potential of this virus to overcome selective pressures exerted by DAAs. The emergence and propagation of antiviral resistance can potentially decrease drug efficacy, as has already been seen for DAAs targeting other viruses, such as HCV, human immunodeficiency virus or influenza virus [17,18,19]. Resistance of SARS-CoV-2 to the protease inhibitor nirmatrelvir, which also has been approved for the treatment of COVID-19 [14,15], has already been reported [20]. Therefore, it is of great importance to identify which mutations confer SARS-CoV-2 resistance to remdesivir, permitting active surveillance of drug resistance-associated substitutions (RAS). Indeed, it appears that SARS-CoV-2 can follow multiple evolutionary pathways towards drug resistance, according to the multiple studies describing unique SARS-CoV-2 remdesivir putative RAS [21,22,23,24,25,26,27,28,29,30,31,32,33,34,35,36,37,38,39]. However, differences in the methods used to assess remdesivir resistance have resulted in varying resistance levels for the same substitutions across studies, which complicates the comparison and categorization of RAS. Additionally, some studies did not include reverse genetic analyses or relied on surrogate models instead of isogenic SARS-CoV-2 reverse genetic systems, which allow for the effect of RAS on both viral replication—the primary target of remdesivir—and propagation to be studied. Furthermore, many of these studies did not investigate the effect of resistance substitutions in vivo.

In this study, we aimed to perform remdesivir resistance selection experiments using an ancestral SARS-CoV-2 isolate and Vero E6 cells. We extensively characterized the genotype and phenotype of viral variants with decreased drug susceptibility, which led to the identification of novel RAS in nsp12 and nsp14. Using an isogenic replicative reverse-genetics system, we performed head-to-head comparisons of the level of drug resistance of previously reported and our newly identified remdesivir nsp12-RAS, thus creating a comprehensive system to categorize the emerging array of SARS-CoV-2 remdesivir RAS.

## 2. Materials and Methods

### 2.1. Virus Cell Culture 

All experiments were performed using the previously described viral stock of the SARS-CoV-2/human/Denmark/DK-AHH1/2020 isolate (GenBank accession no. MZ049597) [10], referred to as the original virus. All work was carried out under biosafety conditions in agreement with Danish regulations and with permission from the Danish authorities, as described in earlier studies [10,20,40,41]. Vero E6, A549 (Sigma, St. Louis, MO, USA), A549-hACE2 (InvivoGen, San Diego, CA, USA) and Calu-3 cells were cultured as previously described [10], in Dulbecco’s modified Eagle’s medium (DMEM, Thermo Fisher, Waltham, MA, USA), DMEM/nutrient mixture F-12 (Thermo Fisher) and Minimum Essential Medium Eagle (EMEM, Thermo Fisher), respectively, supplemented with 10% heat-inactivated fetal bovine serum (FBS, Sigma), 100 U/mL penicillin, and 100 mg/mL streptomycin (Sigma). A549-hACE2 cells were further supplemented with 0.5 µg/mL of puromycin (InvivoGen).

### 2.2. Generation of the RDV Escape Virus

For the remdesivir resistance selection experiment, 1 × 10^6^ Vero E6 cells were infected with the original virus and treated with increasing drug concentrations for three consecutive passages (passages 1, 2, and 3). A non-treated infected control culture was maintained in parallel. Cultures were monitored for the presence of cytopathic effect (CPE) using an inverted light microscope, sub-cultured every 2–3 days according to cell density and supplemented with only media or media containing remdesivir (GS-5734, Acme Bioscience, Palo Alto, CA, USA). Culture supernatants were harvested regularly and stored at −80 °C until further analysis. Details on viral infection and treatment for the different passages can be found in the Supporting Methods.

Drug-free stocks of the non-treated virus control from passage 3 (RDV untreated virus) and treated viruses from passages 1 and 2 (RDV escape passages 1 and 2 viruses, respectively) were prepared by infecting 2.5 × 10^6^ Vero E6 cells with 250 µL of culture supernatant harvested at day 6 (RDV untreated), 80 (RDV escape passage 1), or 14 (RDV escape passage 2) post-infection. The stock of the treated virus from passage 3 (RDV escape virus) was prepared by infecting 5 × 10^6^ Vero E6 cells with 500 µL of culture supernatant harvested at day 18 post-infection. Virus stocks consisted of filtered (0.45 µm filter, Sartorius, Göttingen, Germany) supernatants harvested at day 2 or 3 post-infection that were aliquoted and stored at −80 °C.

### 2.3. Reverse Genetics Experiments

Mutations were introduced into a SARS-CoV-2 subgenomic reporter replicon system based on the SARS-CoV-2/human/Denmark/DK-AHH1/2020 isolate sequence (referred to as the original replicon) [42] using an In-Fusion PCR-based-technique (see details in the Supporting Methods) [43]. Downstream cloning in NEB^®^ 10-beta Competent *E. coli* (New England Biolabs, Ipswich, MA, USA) and further preparation of plasmids using QIA Spin miniprep and Large-Construct kits (Qiagen, Hilden, Germany) were performed following the manufacturer’s instructions [42]. Plasmid sequences were confirmed by next-generation sequencing (NGS) analysis, as described below. Plasmids (4 µg) were then linearized with FastDigest NotI (Thermo Fisher), purified with the Zymo DNA clean & concentrator-25 kit with Zymo-Spin™ IC-XL columns (ZymoResearch, Irvine, CA, USA), and further in vitro transcribed using the mMESSAGE mMACHINE T7 Transcription Kit (Thermo Fisher) according to manufacturer’s instructions [42].

Mutations were introduced into a full-length SARS-CoV-2 infectious system based on the SARS-CoV-2/human/Denmark/DK-AHH1/2020 isolate (referred to as the original clone) [42] using a megaprimer PCR-based technique (see details in the Supporting Methods) [42,43]. Downstream cloning and in vitro transcription were performed as described above. RNA transcripts (1 µg) were transfected into 2 × 10^5^ Vero E6 cells (seeded in 12-well plates) using lipofectamine 2000 (Thermo Fisher) (see details in the Supporting Methods). Cultures were monitored and supernatants harvested as described above. Viral passages were performed by infecting 2 × 10^6^ Vero E6 cells with 250 µL of culture supernatant from day 6 post-transfection, and viral stocks were prepared from the passages as described above.

### 2.4. Determination of Viral Infectivity Titers

Viral infectivity titers were determined by TCID_50_ assays [10]. In brief, 1 × 10^4^ Vero E6, A549-hACE2 or Calu-3 cells were seeded in 96-well plates (Thermo Fisher) and infected the following day with 10-fold serially diluted culture supernatant. After 72 h, plates were processed for immunostaining [10], using SARS-CoV-2 spike chimeric monoclonal primary antibody (no. 40150-D004, Sino Biological, Beijing, China) and F(ab’)2-goat anti-human IgG Fc cross-adsorbed secondary antibody conjugated to horseradish peroxidase (no. A24476, Invitrogen, Waltham, MA, USA). Plates were scanned using an ImmunoSpot series 5 UV analyzer (Cellular Technology limited, CTL, Cleveland, OH, USA), and the score of infected wells for each dilution (tested in quadruplicates) was used to calculate Log_10_TCID_50_/mL following the Reed and Muench method [44]. Viral 50% cytopathic effect titers (CPE_50_) were determined in Vero E6 cells using CPE assays [10], conducted similarly to TCID_50_ assays but performed in 96-well white/clear-bottom plates (Thermo Fisher). After 72 h, CPE was evaluated using the viral ToxGlo assay (Promega, Madison, WI, USA), following the manufacturer’s instructions. Relative light units (RLU), which reflected the number of viable cells, were obtained for each viral dilution (tested in quadruplicates) and normalized to non-infected controls (tested in 24 replicates). Dilutions exhibiting cell viability below 90% were classified as positive for CPE, and the Log_10_CPE_50_/mL was calculated using the Reed and Muench method [44]. The lower limit of quantification (LLOQ) was defined by the lower titer (2.2 Log_10_TCID_50_/mL or Log_10_CPE_50_/mL) that could be calculated from the lowest viral dilution used in the assays (1/10).

### 2.5. Antiviral Activity Assays

Remdesivir, GS-441524, obeldesivir, and molnupiravir (obtained from MedChemExpress, Monmouth Junction, NJ, USA) were diluted in DMSO prior to use.

Drug antiviral activity on infectious viruses was assessed using short-term concentration response assays measuring viral propagation in Vero E6, A549-hACE2 or Calu-3 cells, which permitted the determination of the effective drug concentration as 50% (EC_50_) and 90% (EC_90_), as previously described [10,45]. In brief, 1 × 10^4^ cells were seeded in 96-well plates, and the following day they were infected and treated simultaneously with 2-fold drug dilutions in culture media. For the infection, MOIs ranging from 0.5 to 0.00005 (calculated using infectivity titers determined in the corresponding cell line) were selected based on virus propagation kinetics. After 48 h, plates were processed for immunostaining and scanning as described above. Plates were further analyzed by the automated counting of single SARS-CoV-2 spike protein-positive cells using ImmunoSpot-BioSpotTM 5.0 software (CTL). The mean count of 12-replicate non-infected wells was subtracted from the counts of infected wells, and the values obtained from treated wells (4 replicates) were normalized to untreated controls (4 replicates). EC_50_ and EC_90_ values were calculated in GraphPad prism (version 9.2.0) from the non-linear regression analysis using the formula Y = bottom + (top − bottom)/(1 + 10^(Log10EC50/90−X)xHill Slope^).

In addition, remdesivir antiviral activity on infectious viruses was further evaluated using a CPE reduction assay in Vero E6 cells. CPE reduction assays were conducted similarly to concentration–response assays based on viral propagation. Cells were infected at an MOI of 0.05 for all viruses (calculated using CPE titers) and treated simultaneously with 1.5-fold drug dilutions in culture media. After 72 h, CPE was evaluated as described above. For all viruses, cell viability in non-treated controls ranged from 22% to 33%. The RLU counts of treated wells (4 replicates) were normalized in GraphPad prism, setting the average RLU value of 12 non-infected wells as 0% and the average RLU value of 4 non-treated wells as 100%. Virus yield assays were performed by inoculating 1 × 10^6^ Vero E6 cells with the specified viruses at an MOI of 0.01 (calculated using infectivity titers). Immediately after, 10 µM of remdesivir was added to treated cultures, which was replenished daily. Untreated cultures were kept in similar conditions. Supernatants were harvested daily and stored at −80 °C for viral titrations assays as described above.

As remdesivir is a nucleotide analog inhibiting viral replication, we also investigated the effect of the drug using replication assays. Using subgenomic replicons, we determined the fold-change in replication levels in the presence of remdesivir compared to replication levels in untreated cells. For that, RNA transcripts (0.5 µg) were transfected into Vero E6 or A549 cells as described above (see details in the Supporting Methods) [42]. Reporter luciferase activity was measured using the NanoGlo^®^ Luciferase Assay System (Promega) at 1 h and 24 h post-transfection, following the manufacturer’s instructions. Cell lysates from each transfected 12-well were transferred into 3 wells (technical replicates) of 96-well white plates (Thermo Fisher) for the measurement of luciferase activity in a Synergy LX MultiMode Microplate Reader (Biotek, Winooski, VT, USA). The mean count of 6 empty wells was subtracted from the counts of wells containing cell lysate, and the values from the 3 wells of the 1 h timepoint were subtracted from the counts of the 3 wells from the 24 h timepoint. Values were multiplied by 3 to express luciferase activity as relative light units per transfection well (RLU/well). The change in luciferase activity from 0 µM to 25 µM (Vero E6) or 1 µM (A549) of remdesivir was calculated for all replicon clones. The fold-change in the replication of each mutant compared to the original replicon in the presence of remdesivir was then calculated by dividing the change in luciferase activity of each mutant replicon by the corresponding value obtained for the original replicon.

### 2.6. Growth Kinetics and Competition Assays for the Determination of Viral Fitness

The production of infectious viruses and cell propagation were evaluated by infecting 2 × 10^5^ Vero E6 cells (seeded in 12-well plates) or 1 × 10^4^ Vero E6 cells (seeded in 96-well plates), respectively, at MOI 0.1 and 0.01 (calculated using infectivity titers). Culture supernatants were harvested at 24 h and 48 h post-infection, stored, and further used to determine viral infectivity titers with TCID_50_ assays. For assessing viral propagation, intracellular immunostaining detecting virus antigen-positive cells was performed at 24 h and 48 h post-infection, as described above. The mean count of 6-replicate non-infected wells was subtracted from the counts of infected 4-replicate wells. Competition assays were performed by co-infecting 2 × 10^5^ Vero E6 cells (seeded in 12-well plates) with the original and the RDV escape viruses at two different MOI ratios, as indicated. Culture supernatants from day 3 post-infection were analyzed by NGS. The frequency of nsp12-E796D (frequency > 99% in the RDV escape virus and undetectable in the original virus) was used to estimate the relative frequency of each virus in the viral population.

### 2.7. In Vivo Experiments

Animal experiments were conducted in certified animal facilities at Statens Serum Institut, Denmark, under animal study proposal 2020-15-0201-00718, approved by the Danish Animal Experiments Inspectorate. All animals were housed in individually ventilated cages with food and water ad libitum on a 12 h light–dark cycle. All experimentation was conducted during the light cycle and consistent with affirmative responses to the Arrive-10 questionnaire. Five-to-seven-week-old male Syrian hamsters *Mesocricetus auratus* (Janvier, Le Genest-Saint-Isle, France) were distributed in 3 groups and infected by nasal inoculation with 1 × 10^4^ TCID_50_ units in 100 µL of transport medium (DMEM supplemented with 10% FBS and 100 U/mL of penicillin, 100 mg/mL of streptomycin, and 0.25 mg/mL of amphotericin B Gibco (Waltham, MA, USA)) of either original virus (*n* = 4), nsp12-E796D mutant virus (*n* = 4), or inoculated with 100 µL of transport medium (non-infected control, *n* = 4) at day post-inoculation (dpi) 0. The general health/disease status of the animals following infection was monitored using body weight from dpi 0 (prior to infection) to the scheduled experimental endpoint, dpi 5. Oropharyngeal swabs were collected daily from dpi 0 (prior to infection) to dpi 5 in 400 µL of transport medium and stored at −80 °C. Samples were then thawed, clarified by centrifugation, and mixed with 3 volumes of Trizol LS (Thermo Fisher) for viral RNA analysis (see below). Hamster blood samples were collected into EDTA containing sample tubes upon euthanasia at dpi 5. Plasma was clarified by centrifugation, stored at −80 °C and further used to determine neutralizing titers (see below). Lungs were collected upon euthanasia at dpi 5 and processed for the histopathological analysis and determination of viral RNA titers as described below.

### 2.8. Lung Tissue Processing

Upon euthanasia of hamsters at dpi 5, the right lung from each animal was immersion fixed in 10% buffered formalin and processed for histopathological analysis, as previously described [46], with minor modifications (see details in the Supporting Methods). The pathologists performing the histological examination were blinded with respect to the origin of the experimental groups, and lesions were scored as absent or present (−/+) or as absent, few, or numerous (−/+/++). The left lung from each animal was processed to determine viral RNA titers. Upon euthanasia, lungs were cut into pieces of approximately 4 × 4 mm and stored in 5 mL of RNAlater (Sigma) at −80 °C until further use. Thawed tissue was depleted of liquid with sterile adsorbent swabs and homogenized in 1 mL of Trizol (Thermo Fisher) using a MagNA Lyser instrument (Roche, Basel, Switzerland) at 6500 rpm for 20 s in Magna Lyser green beads tubes (Roche). Viral RNA was extracted from the lung homogenate as described below.

### 2.9. Determination of Viral RNA Titers

Viral RNA was extracted through the addition of chloroform and phase separation followed by purification on a Zymo RNA clean & concentrator-5 or 25 kits (ZymoResearch), following the manufacturer’s instructions. Virus RNA titers were determined by reverse-transcription quantitative PCR (RT-qPCR), as previously described [42], using the TaqMan Fast Virus 1-Step Master Mix (Thermo Fisher). RNA standards ranging from 1 × 10^1^ to 1 × 10^5^ RNA copies/mL (Twist Bioscience, San Francisco, CA, USA), a negative control, and diluted samples were included as two technical replicates in each analysis, run on a Lightcycler 96 (Roche) and analyzed with the Lightcycler 96 software version 1.10.1320 (Roche). The LLOQ was calculated as the average value of the non-infected control animals plus three times its standard deviation, which was 0.5 RNA copies/µL for daily oropharyngeal swabs and 0.01 RNA copies/ng of total RNA for lung homogenates.

### 2.10. Neutralization Assay

Neutralization of the original virus was performed as previously described [10,47]. In brief, 1 × 10^4^ Vero E6 cells were seeded in 96-well plates. The next day, the original virus (MOI of 0.02, calculated using infectivity titers) was incubated for 1 h at room temperature (RT) with serially diluted, heat-inactivated (at 56 °C for 30 min) hamster plasma (1/100 to 1/51,200 dilution) at a 1:1 ratio and subsequently added to the cells (4 replicates). Cells were processed for immunostaining, scanning, and automated counting, as described above, 48 h after infection. Plasma 50% inhibitory dose (ID_50_) values were calculated in GraphPad prism using the same formula described above to obtain EC_50_ values. The LLOQ was defined by the lowest dilution used in the assay (1/100). A 1/800 dilution (1.25 µg/mL) of a mouse-derived SARS-CoV-2 spike neutralizing antibody (RRID: AB_2857935, Sino Biological) was used as positive control, giving an average of 77% neutralization.

### 2.11. Next-Generation Sequencing Analysis

Sequence analysis of the nearly full-length SARS-CoV-2 genome was performed as previously described [10]. Briefly, RNA was extracted as described above and five overlapping amplicons covering the nearly full-length viral genome were obtained after a reverse transcription PCR (RT-PCR). The five amplicons were then subjected to library preparation using the NEBNext Ultra II FS DNA Library Prep Kit (New England Biolabs). NGS was conducted in-house by Illumina Miseq (Illumina, San Diego, CA, USA) as previously described [48], with minor modifications [10]. Details on the processing and sequencing of the overlapping amplicons can be found in the Supporting Methods.

### 2.12. In Silico Structural Analysis of Remdesivir Resistance-Associated Substitutions

The PyMOL 2.5.0 [49] software was used to show the different nsp12 substitutions in the nsp7-nsp8-nsp12 structure (PDB entry: 7UO4 [50]), and to perform in silico mutagenesis of E796D. To assess the dynamics of motif D in nsp12, we analyzed a 10 µs molecular dynamics simulation of the nsp7-nsp8-nsp12 complex (PDB entry: 6M71) [51], made available by D. E. Shaw Research [52], under the Creative Commons Attribution 4.0 International Public License. A principle component analysis of the simulation was performed using the ProDy software [53] and based on amino acid residues 780–803 in nsp12-motif D. Calculations of 4 modes were performed based on heavy atoms. Residues interacting with amino acids E796 or K798 throughout the simulations were identified by calculating residue–residue distances with the Visual Molecular Dynamics 1.9.4 software [54].

### 2.13. Statistical Analysis

Data was plotted and a statistical analysis was performed in GraphPad prism. The *p* values and specific statistical tests performed for each experiment are indicated in the figure legends. Data was analyzed using unpaired parametric tests with no assumption about equal standard deviations. Unpaired *t* tests with Welch correction were used to compare the means between two samples and multiple unpaired *t* tests with Welch correction were used to compare the means of two samples when multiple comparisons were made simultaneously. Multiple *t* tests were corrected for multiple comparisons. Statistical significance was defined as a *p* value less than 0.05 in all tests. For viral RNA titers and neutralization data, the non-infected animal group was not included in the statistical analysis due to the samples having values under the LLOQ of the assays.

## 3. Results

### 3.1. Emergence of SARS-CoV-2 Resistance to Remdesivir Following Serial Passage in Vero E6 Cells

**Figure 1:**
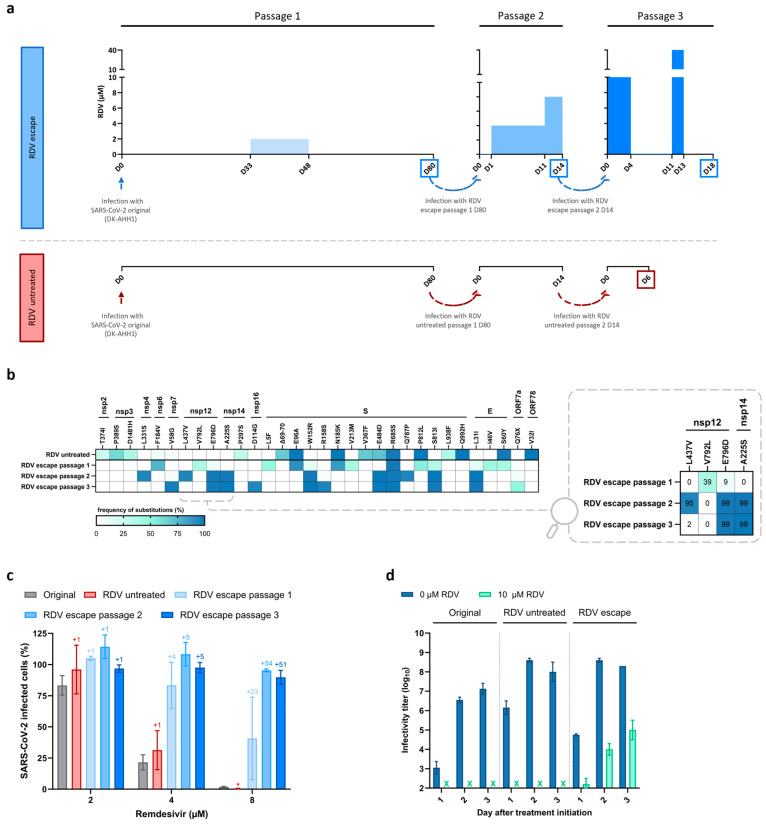
Generation and characterization of remdesivir escape viruses. (**a**) Graphical representation of the experiment for the selection of remdesivir resistance. The RDV escape (**top**) and untreated (**bottom**) viruses were generated in three serial passages (passages 1, 2, and 3), as indicated. Top graphs show at which days after infection (X-axis) the indicated virus was exposed to the specified remdesivir concentrations (Y-axis). Bottom lines show how many days after infection the indicated virus was cultured. Dotted arrows between passages indicate that culture supernatant from the last day of the previous passage was used for the infection of naïve Vero E6 cells in the following passage. Squares indicate at which day after infection the virus was drug-free passaged to produce a viral stock. D, day. (**b**) Genetic characterization of RDV untreated and RDV escape passages 1, 2, and 3 viruses. The heat map shows the frequency (cutoff of ≥20% for at least one virus) of amino acid substitutions present throughout the near-full-length genome sequence of the indicated viruses, compared to the original virus. Substitutions are indicated using protein-specific amino acid numbers, with the proteins indicated on top. Frequencies are displayed using a three-color gradient, with the specific percentages of nsp12 and nsp14 substitutions present in the RDV escape passages 1, 2, and 3 viruses also indicated in a zoomed-in section of the heatmap. (**c**) Antiviral activity of remdesivir in drug concentration–response assays performed in Vero E6 cells. The graph shows the infection levels of different viruses, measured as SARS-CoV-2-infected cells normalized to nontreated controls (Y-axis, in percentage), at different remdesivir concentrations (X-axis, µM). Bars represent the mean of four replicate infections, with error bars indicating the standard error of the mean. Median fold-increases in infection relative to the original virus are indicated above the bars for the different viruses. * Signifies that no infected cells were detected for the RDV untreated virus, preventing the calculation of the median fold-change. Non-linear regression curves of the data are shown in Figure S1. (**d**) Antiviral activity of remdesivir in virus yield assays performed in Vero E6 cells. The graph shows viral infectivity titers in supernatants (Y-axis, Log_10_TCID_50_/mL) of treated (10 µM RDV, in turquoise) and untreated (0 µM RDV, in blue) cultures infected with different viruses (top) after experiment initiation (X-axis, days). Bars represent the mean of two independent experiments (one titration per experiment), with error bars indicating the standard error of the mean. X indicates that values were under the limit of detection (2.2 Log_10_TCID_50_/mL).

We performed remdesivir escape experiments using an ancestral SARS-CoV-2 isolate (DK-AHH1 [10], referred to as the original virus). The original virus was subjected to three long-term passages in Vero E6 cells, where the remdesivir concentration was progressively increased with periods of drug withdrawal (Figure 1a). Similar culture conditions were applied for a non-treated control virus (Figure 1a). The sequence analysis of the viruses obtained during the experiment showed the emergence of mutations throughout the entire genome of both the virus treated with remdesivir (obtained after the third passage and referred to as RDV escape virus) and the non-treated control (referred as to RDV untreated virus) (Table S1). However, only the RDV escape virus acquired unique substitutions in the RdRp domain of nsp12, which is the target of remdesivir. After the first passage at low drug pressure, two nsp12 substitutions emerged, V792L and E796D, both at a frequency <40%. Upon exposure to higher drug concentrations in passage 2, V792L became undetectable, whereas E796D and a new substitution (L437V) became dominant (frequency > 80%) in the viral population. Interestingly, substitution A225S in the exonuclease (ExoN) domain of nsp14, which removes mis-incorporated nucleotides during RNA synthesis, emerged at a frequency >99%. Upon further escalation of the drug concentration during passage 3, the frequency of L437V decreased to <5%, while nsp12-E796D and nsp14-A225S continued to be dominant (Table S1). The viruses obtained at the end of each passage were subjected to a drug-free passage for further genotypic and phenotypic characterization. All observed substitutions were maintained without drug pressure (Figure 1b and Table S1).

In concentration–response assays, the remdesivir-treated viruses showed higher infection levels in the presence of remdesivir compared to both the original and RDV untreated viruses (Figure 1c), with the largest differences exceeding 20-fold at 8 µM of remdesivir. As a result, the remdesivir-treated viruses exhibited increased effective drug concentration 50% (EC_50_) and 90% (EC_90_) values compared to the original virus (Figure S1a), with the RDV escape (passage 3) virus showing the greatest changes (3.7- and 2.9-fold increases in EC_50_ and EC_90_, respectively). Moreover, in a 3-day virus-yield reduction experiment with a fixed remdesivir concentration of 10 µM, cultures infected with the original or RDV untreated viruses showed no signs of CPE nor detectable viral infectivity titers (50% tissue culture infectious doses/mL; TCID_50_/mL) in the supernatant during treatment. In contrast, in the culture infected with the RDV escape virus, CPE and high infectious titers were detected throughout the experiment, indicating a significant overall loss of remdesivir susceptibility (Figure 1d).

Thus, the combination of decreased drug susceptibility and emergence of substitutions in the drug target strongly suggested that the RDV escape virus had indeed acquired remdesivir resistance. Moreover, compared to the original virus, the RDV escape virus exhibited increased EC_50_ values for the remdesivir parent nucleoside GS-441524 (3.1-fold), as well as the oral prodrug obeldesivir (3.4-fold) (Figure S1b,c), which have shown antiviral efficacy against SARS-CoV-2 in vitro and in vivo [55,56]. However, no major differences in EC_50_ values were observed for the broad-spectrum nucleotide analog molnupiravir (Figure S1d).

### 3.2. Substitution nsp12-E796D Reduces Remdesivir Susceptibility

**Figure 2:**
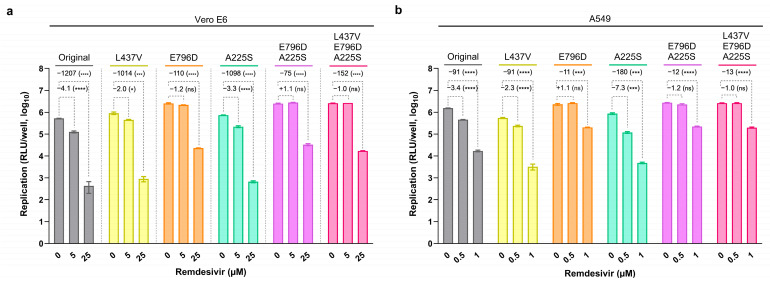
Remdesivir susceptibility of the original and mutant subgenomic replicons in replication assays performed in (**a**) Vero E6 and (**b**) A549 cells. Graphs show the luciferase activity (Y-axis, log_10_ relative light units [RLU]/well) at 24 h post-transfection of the different replicon clones (top) with different concentrations of remdesivir (X-axis, µM). Bars represent the mean luciferase reads from six replicates, with error bars indicating the standard error of the mean (* *p* = 0.332; *** *p* = 0.0002; **** *p* < 0.0001; ns, not significant. Multiple unpaired *t* test with Welch correction). For each replicon clone, the median fold-reduction (−) or increase (+) in replication from the non-treated control to the treatment with different concentrations of remdesivir is indicated on top of the dotted dashes.

To investigate the role of the nsp12 and nsp14 substitutions observed at high frequencies during the remdesivir-resistance selection experiment, we reverse-engineered substitutions nsp12-L437V, nsp12-E796D, and nsp14-A225S, singly and in different combinations, into a subgenomic SARS-CoV-2 reporter replicon clone of isolate DK-AHH1 (referred to as the original replicon) [42]. Viral replication assays were performed in Vero E6 cells to assess the effect of these substitutions on remdesivir susceptibility by measuring replication activity in the presence of 5 or 25 µM of remdesivir. In the absence of drug, the original and all mutant replicons exhibited high levels of replication, whereas all showed a concentration-dependent inhibition of replication in the presence of remdesivir (Figure 2a). Compared to the original replicon, all mutants harboring substitution nsp12-E796D exhibited significantly higher replication levels in the presence of 25 µM of remdesivir, indicating decreased drug efficacy. The nsp12-E796D substitution alone conferred a 11.0-fold increase in replication compared to the original replicon, and the highest increase in replication was observed for the combination of nsp12-E796D with nsp14-A225S (16.1-fold). Similar results were observed in replication assays performed in A549 cells (Figure 2b), where mutants E796D and E796D+A225S exhibited an 8.3- and 7.6-fold increase in replication compared to the original replicon, respectively, in the presence of 1 µM of remdesivir. Thus, substitution nsp12-E796D was associated with reduced remdesivir susceptibility, particularly in combination with nsp14-A225S.

**Figure 3:**
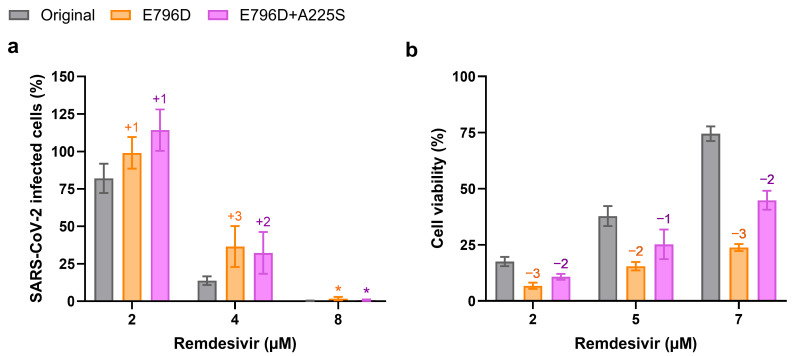
Antiviral activity of remdesivir against the original and mutant viruses in (**a**) drug concentration–response or (**b**) cytopathic effect reduction assays performed in Vero E6 cells. (**a**) The graph shows infection levels of different viruses, measured as SARS-CoV-2-infected cells normalized to nontreated controls (Y-axis, in percentage), at different remdesivir concentrations (X-axis, µM). Bars represent the mean of four replicate infections, with error bars indicating the standard error of the mean. Median fold-increases in infection relative to the original virus are indicated above the bars for the different viruses. * Indicates that no infected cells were detected for the original virus, preventing the calculation of the median fold-change. Non-linear regression curves of the data are shown in Figure S1. (**b**) Cell viability in cultures infected with different viruses, measured in relative light units (RLU), which are proportional to the number of viable cells. The graph shows RLU normalized to nontreated controls (Y-axis, in percentage), at different remdesivir concentrations (X-axis, µM). Bars represent the mean of four replicate infections, with error bars indicating the standard error of the mean. Median fold-reductions in cell viability relative to the original virus are indicated above the bars for the different viruses.

To evaluate the effect of nsp12-E796D on remdesivir susceptibility within the complete SARS-CoV-2 life cycle, we engineered nsp12-E796D, both individually and in combination with nsp14-A225S, into an infectious clone of isolate DK-AHH1 (referred to as the original clone) [42]. Both mutants were viable in Vero E6 cells, maintaining the introduced mutations after passage, with no further acquisition of substitutions with frequency > 5% in either nsp12 or nsp14 (Table S2). In concentration–response assays conducted in Vero E6 cells, both E796D and E796D+A225S mutants showed higher infection levels compared to the original virus at several concentrations of remdesivir (Figure 3a). Consistently, in CPE reduction assays performed in Vero E6 cells, both mutants showed reduced recovery of cell viability compared to the original virus at various remdesivir concentrations (Figure 3b). However, the differences in EC_50_ compared to the original virus were low, with only a 1.1- and 1.3-fold increase in EC_50_ values for the E796D and E796D+A225S mutants, respectively (Figure S1e). Similar results were observed in Calu-3 and A549-hACE2 cells, where neither mutant reproduced the resistance phenotype of the RDV escape virus. However, the EC_50_ values compared to the original virus were slightly higher in these cell lines, particularly for the E796D+A225S mutant in A549-hACE2 cells (Figure S1f,g). For comparison, we generated a virus carrying nsp12-RAS S759A+V792I, which showed the highest EC_50_ fold-change in previous studies [21]. In Vero E6 cells, compared to the original virus, the S759A+V792I virus exhibited an 11.7-fold increase in EC_50_, thus conferring a 9.0-fold higher resistance than E796D+A225S (Figure S1h).

### 3.3. Viral Fitness Might Contribute to Remdesivir Resistance in Vero E6 Cells

**Figure 4:**
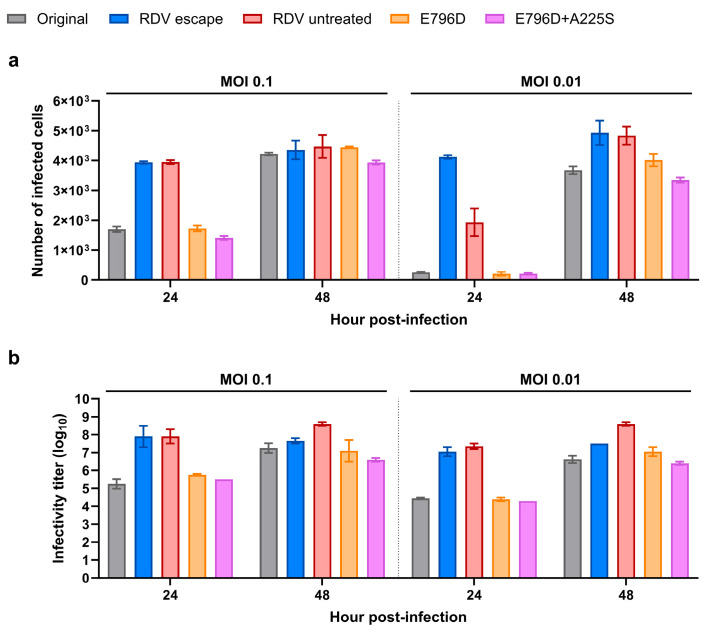
Viral growth kinetics in Vero E6 cells expressed as (**a**) number of total infected cells and (**b**) infectivity titer for the different viruses. (**a**) The graph shows the total number of SARS-CoV-2 antigen-positive cells (Y-axis) after infection (X-axis, hours) at the indicated MOI (top). Bars represent the mean of four replicates for all viruses except for the original virus, for which the mean of eight replicates is represented. Error bars represent standard error of the mean. (**b**) The graph shows viral infectivity titers (Y-axis, Log_10_TCID_50_/mL) after infection (X-axis, hours) at the indicated MOI (top). Bars represent the mean of two independent experiments (one titration per experiment) for all viruses except for the original virus, for which four independent experiments (one titration per experiment) are represented. Error bars represent standard error of the mean.

Our results clearly suggest that nsp12-E796D causes remdesivir resistance by enhancing viral replication in the presence of the drug in replication assays. However, the lack of significant EC_50_ and EC_90_ changes observed in infectious assays with the mutant viruses suggest that additional factors, beyond the effect that nsp12-E796D and nsp14-A225S have on replication, likely contribute to the resistance phenotype observed on the RDV escape virus (which exhibited a significant increase in EC_50_ value in Vero E6 cells, Figure S1a). Hence, we investigated whether changes in viral fitness correlated with the different drug susceptibilities observed above. In competition assays where cells were co-infected with either a 1:1 or 10:1 infectious ratio of the original–RDV escape viruses, we observed an increased frequency of the SNP associated with the RDV escape virus in both ratios at 72 h post-infection, indicating a relatively higher fitness of the RDV escape virus compared to the original virus (Figure S2). Furthermore, in growth kinetics experiments measuring either viral propagation (number of infected cells in the culture, Figure 4a) or secretion of infectious viral particles (TCID_50_/mL in the supernatant, Figure 4b), cultures infected with the RDV escape virus exhibited a higher number of infected cells and infectivity titers as early as 24 h post-infection when compared to the original virus, regardless of the MOI used, which further supports the enhanced fitness of the RDV escape virus (Figure 4a,b). This enhanced fitness phenotype of the RDV escape virus was not recapitulated by the specific substitutions involved in drug resistance. Neither E796D nor E796D+A225S mutants differed in growth kinetics from the original virus, exhibiting an overall similar number of infected cells and infectivity titers (Figure 4a,b). Hence, we also investigated the fitness of the RDV untreated virus, which was cultured in parallel to the RDV escape virus during the resistance selection experiment. Similarly to the RDV escape virus, the RDV untreated virus exhibited a higher number of infected cells and infectivity titers compared to the original virus (Figure 4a,b). Thus, the RDV untreated virus also exhibited a fitness enhancement, indicating that the superior fitness of the RDV escape virus could be related to its adaptation to Vero E6 cells, which led to the emergence of several substitutions throughout the genome of both RDV escape and RDV untreated viruses, most notably on the spike protein (Figure 1b and Table S1).

Taken together, these results suggest that the enhanced fitness of the RDV escape virus (mostly driven by non-RAS substitutions resulting from culture adaptation) might have contributed to the higher remdesivir EC_50_/EC_90_ values exhibited by this virus in Vero E6 cells, compared to the modest increase observed for the mutants (Figure S1a,e). However, the increased fitness observed in the RDV untreated control virus did not result in reduced remdesivir susceptibility (Figure 1c,d and Figure S1a), indicating that both the E796D+A225S substitutions and enhanced fitness may contribute to the remdesivir resistance observed for the RDV escape virus in Vero E6 cells.

### 3.4. Substitution nsp12-E796D Does Not Alter SARS-CoV-2 Viability In Vivo

**Figure 5:**
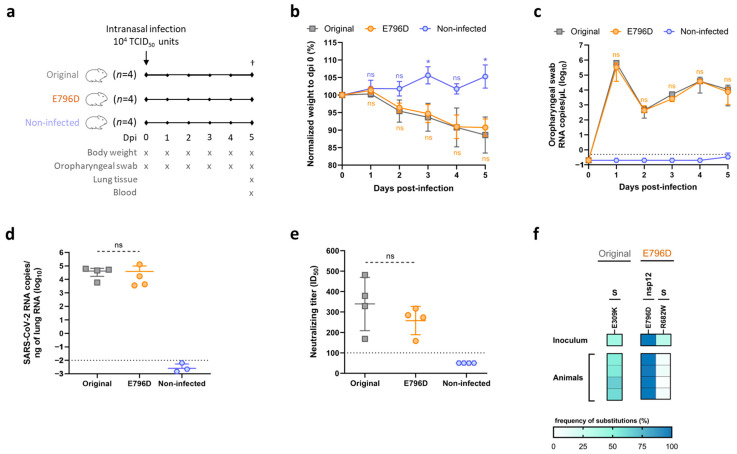
Effect of nsp12-E796D in vivo. (**a**) Animal experiment outline. Hamsters were inoculated intranasally with the different viruses or transport media (non-infected) (*n* = 4 for each group) at day 0 post-infection (dpi). All animals were euthanized (†) at the endpoint of the experiment (dpi 5). An overview of the different measurements and samples taken at the specific timepoints is shown. (**b**) Animal body weight. The graph shows the body weight of the different animal groups normalized to their respective weight at dpi 0 (Y-axis, in percentage) after infection (X-axis, days). Symbols represent the mean of four animals, and error bars represent standard deviation. Statistical comparisons were conducted between original infected animals and E796D infected animals or non-infected animals (* *p* = 0.0332; ns, not significant. Multiple unpaired *t* test with Welch’s correction). (**c**) RNA viral titers in oropharyngeal swabs. The graph shows SARS-CoV-2 RNA copies/µL (Y-axis, in log_10_) for the different animal groups after infection (X-axis, days). Symbols represent the mean of four animals, and error bars represent standard deviation. Statistical comparisons were conducted between original and E796D infected animals (ns, not significant. Multiple unpaired *t* test with Welch’s correction). The dotted line indicates the LLOQ. (**d**) RNA viral titers of lung homogenates. The graph shows SARS-CoV-2 RNA copies/ng of total RNA (Y-axis, in log_10_) for the different animal groups (X-axis) from lung tissue samples collected upon euthanasia. Symbols represent individual values and group means are shown as horizontal bars with standard deviation (ns, not significant. Unpaired *t* test with Welch’s correction). The dotted line indicates the LLOQ. (**e**) Neutralization levels of the original virus by animal plasma samples. The graph shows neutralizing titers, measured as ID_50_ and calculated from the non-linear regression curves in Figure S3 (Y-axis), of plasma samples collected upon euthanasia from the different animal groups (X-axis). Symbols represent individual values and group means are shown as horizontal bars with standard deviation (ns, not significant. Unpaired *t* test with Welch’s correction). The dotted line indicates the LLOQ. (**f**) Genetic stability of original and E796D mutant viruses after animal infection. Heat maps show the frequency (analyzed by NGS) of amino acid substitutions present through the near full-length genome sequence of the viruses recovered from oropharyngeal swabs collected on the day of euthanasia from animals infected with the original (**left**) or E796D mutant (**right**) viruses. The frequency of the substitutions in the viral inoculum used to infect the animals at dpi 0 is also indicated. The original virus isolate (GenBank MZ049597) was used as reference sequence, with a cutoff of ≥20%. Substitutions are indicated using protein-specific amino acid numbers, with the proteins indicated on top. Frequencies are displayed with a three-color gradient.

To evaluate the effect of substitution E796D in vivo, we performed infection experiments in Syrian hamsters, which are susceptible to SARS-CoV-2 and develop clinical disease upon infection. These experiments assessed viral fitness and genetic stability in vivo, but did not evaluate the impact of E796D on resistance to remdesivir. Hamsters were inoculated intranasally with 10^4^ TCID_50_ units of the original virus or the E796D mutant. In parallel, a non-infected control group was inoculated with a corresponding volume of media (Figure 5a). In contrast to the non-infected animals, which increased in body weight, the hamsters infected with the original virus or with the E796 mutant virus developed clinical disease in a similar manner, exhibiting a progressive body weight reduction until the endpoint of the experiment at day 5 post-infection (Figure 5b). Moreover, both original and E796D mutant infected animals exhibited a similar evolution of SARS-CoV-2 RNA titers in oral swabs over time (Figure 5c). The SARS-CoV-2 RNA titers in lung tissue were also similar between the two groups, with average values of 4.5 and 4.1 log_10_ RNA copies/ng of total RNA for original and E796D mutant infected animals, respectively (Figure 5d). Tissue from the lungs was subjected to histopathological investigation. Whereas no pathological changes were observed in the non-infected animals, both original and E796D mutant infected animals showed a similar pattern of lung lesions (Table S3). We also determined the neutralizing antibody (NAb) titers against the original virus in all animals to evaluate the immune response induced by viral infection. While NAb titers were undetectable for the non-infected animals, original and E796D mutant infected animals exhibited similar neutralizing activity in plasma (Figure S3), with mean ID_50_ values of 339 and 258, respectively (Figure 5e). Moreover, when we analyzed the SARS-CoV-2 sequence recovered from the oral swabs of E796D mutant infected animals at day 5 post-infection, substitution E796D was genetically stable, with a frequency over 99% (Figure 5f). Similarly, no genetic changes were observed in the recovered original virus (Figure 5f).

These results demonstrate that the nsp12-E796D substitution does not impair SARS-CoV-2 viability and remains genetically stable in vivo, highlighting the mutation’s potential to arise and persist in the clinic.

### 3.5. Most nsp12-RAS Confer Moderate Resistance to Remdesivir

**Figure 6:**
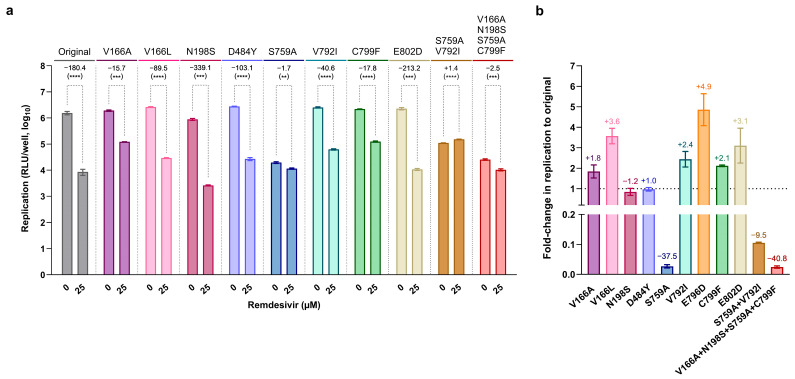
Remdesivir susceptibility and replication levels of mutant replicons harboring different remdesivir RAS. (**a**) Remdesivir susceptibility of the mutant subgenomic replicons harboring previously described SARS-CoV-2 RAS in replication assays performed in Vero E6 cells. The graph shows the luciferase activity (Y-axis, log_10_ relative light units [RLU]/well) at 24 h post-transfection of the different replicon clones (top) with different concentrations of remdesivir (X-axis, µM). Bars represent the mean luciferase reads from six replicates for all mutant replicons and from eighteen replicates for the original replicon. Error bars indicate the standard error of the mean. (** *p* = 0.0021; *** *p* = 0.0002; **** *p* < 0.0001. Multiple unpaired *t* test with Welch correction). For each replicon clone, the median fold-reduction (−) or increase (+) in replication from 0 to 25 µM of remdesivir is indicated on top of the dotted dashes. (**b**) Replication levels of different mutant subgenomic replicons in assays performed in Vero E6 cells. The graph shows the fold-change in luciferase activity (measured as relative light units [RLU]/well at 24 h post-transfection of 0.5 µM of RNA) compared to the original replicon (Y-axis, in percentage) for the different mutants (X-axis). Bars represent the mean fold-change in luciferase reads from all replicates as indicated in (**a**), with error bars indicating the standard error of the mean. The median fold-reduction (−) or increase (+) in replication compared to the original replicon is indicated above the bars, while the dotted line represents no change in replication.

We employed replicon assays to investigate the impact of different nsp12-RAS on remdesivir susceptibility. We performed head-to-head experiments comparing the remdesivir susceptibility of the original replicon to the susceptibility of nsp12-E796D and 10 previously reported nsp12 remdesivir RAS or combinations of RAS [27,34,36,37]. Thus, we engineered single substitutions V166A, V166L, N198S, D484Y, S759A, V792I, C799F, or E802D into the DK-AHH1 subgenomic replicon clone. Additionally, since substitution S759A was originally described in combination with V792I or V166A, N198S, and C799F, we also generated S759A+V792I and V166A+N198S+S759A+C799F mutants. We then determined their replication activity in the presence of 25 µM of remdesivir in Vero E6 cells (Figure 6a). Compared to their corresponding non-treated controls, all single mutants exhibited a significant decrease in replication in the presence of remdesivir, except for S759A, for which replication was much less affected, indicating higher levels of drug resistance for this RAS.

Next, we grouped the substitutions into three categories according to their effect on replication in the presence of remdesivir compared to the original replicon (Figure 6a). Substitutions N198S, D484Y, and E802D conferred no detectable levels of remdesivir resistance (<2-fold), with replication differences of 0.5-, 1.7-, and 0.8-fold compared to the original replicon. Substitutions V166A, V166L, V792I, and C799F conferred 11.5-, 2.0-, 4.4-, and 10.1-fold increased replication compared to the original replicon. Hence, these substitutions and E796D showed intermediate resistance (2- to 20-fold). Substitution S759A exhibited high resistance (>20-fold). In fact, S759A conferred the highest levels of remdesivir resistance, with the single mutant exhibiting a 106.1-fold increase in replication, which was further increased in the double mutant S759A+V792I (252.6-fold). The mutant V166A+N198S+S759A+C799F also exhibited high levels of resistance (72.7-fold). Thus, in this assay, compared to the resistance observed for E796D+A225S, resistance levels for the combinations containing S759A (S759A+V792I and V166A+N198S+S759A+C799F) were 15.7- and 4.5-fold higher, respectively.

Regarding the effect of the different substitutions on SARS-CoV-2 replication in the absence of remdesivir (Figure 6b), no negative effect was observed for nsp12-RAS causing no detectable to intermediate resistance. In fact, the E796D mutant exhibited a 4.9-fold increase in replication compared to the original replicon. For the previously described nsp12-RAS V166A, V166L, V792I, C799F, and E802D, enhanced replication (up to 3.6-fold) compared to the original replicon was also observed, whereas N198S and D484Y showed similar replication levels. The only RAS that negatively affected replication was S759A, causing a 37.5-fold decrease in replication. The combination of S759A with V792I, although able to improve replication, did not substantially restore the levels observed for the original replicon.

### 3.6. In Silico Structural Analysis Correlates the Location of nsp12-RAS with Levels of Remdesivir Resistance

**Figure 7:**
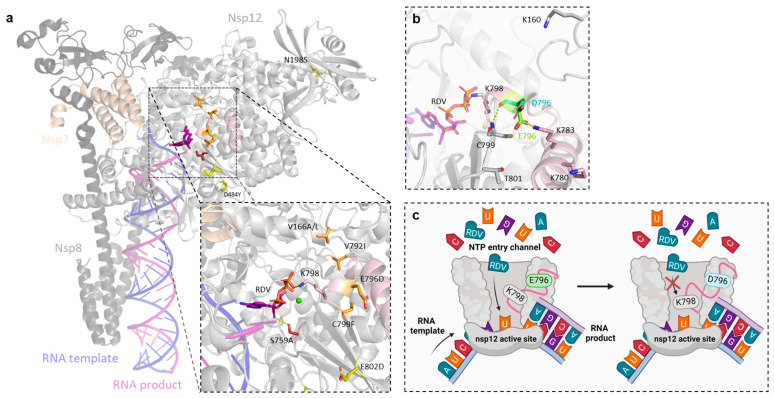
Structural analysis and modeling of SARS-CoV-2 RAS against remdesivir. (**a**) Overview of previously described RAS and nsp12-E796D in the structure of the SARS-CoV-2 nsp7-nsp8-nsp12 complex (PDB entry: 7UO4 [50]). The proteins nsp7 (beige), nsp8 (dark gray), and nsp12 (light gray) are shown together with the RNA template (blue) and product (purple) strands. Motif D (light pink) of nsp12 is also indicated. Substitutions V166A/L, N198S, D484Y, S759A, V792I, E796D, C799F, and E802D are shown as sticks, colored according to their levels of remdesivir resistance: not detectable (yellow), intermediate (orange), or high (red). Residue K798 is also indicated. Dashed yellow lines indicate the interactions of S759 and K798 with RDV in the nsp12 active site. The green sphere indicates a Mg^2+^ ion. (**b**) Predicted structural consequences of E796D in nsp12 (PDB entry: 7UO4 [50]). The original residue E796 (green), the modeled substitution D796 (cyan), and the interaction partners of E796 found in the molecular dynamics simulation analysis (Figure S4) are shown as sticks. Residue K798 is also shown, interacting (yellow dashes) with RDV in the nsp12 active site. (**c**) Potential effect of nsp12-E796D on remdesivir incorporation. The depiction shows the nucleotide triphosphate (NTP) entry channel and active site of nsp12 (light gray), together with the dynamic motif D (light pink) where residues E796 and K798 are located. The RNA template (blue) and product (purple) strands are also shown. Compared with the original residue E796 (in green, left picture), substitutions D796 (in cyan, right picture) could alter the dynamics of motif D, changing the interaction of K798 with RDV and making its incorporation less favorable. Created with BioRender.com.

An in silico structural analysis of the studied SARS-CoV-2 nsp12-RAS suggested that differences in remdesivir resistance levels correlated with the different locations of the substitutions in the protein (Figure 7a). Substitutions N198S and D484Y, which conferred no detectable remdesivir resistance, are located on the surface of the protein and in positions not predicted to affect RNA synthesis. In contrast, S759A, causing the highest remdesivir resistance, is located in the active site. In fact, S759 is a catalytically active amino acid that directly interacts with remdesivir. Other substitutions causing no detectable (E802D) or intermediate (V166A/L, V792I, E796D, and C799F) resistance are adjacent to the active site, clustered around motif D, although, among these, E802D is located the furthest away from the active site. Motif D is a dynamic loop containing K798, which is a catalytic lysine directly involved in nucleotide diffusion into and binding in the active site [57,58]. Consequently, any substitution near motif D that alter its dynamics could indirectly affect remdesivir incorporation.

Residue E796 is located in motif D, in close proximity to K798. By applying principal component analysis of nsp12-motif D in a molecular dynamics simulation of nsp7-nsp8-nsp12, we observed that motif D is indeed dynamic, adapting different conformations, with E796 and K798 being the most dynamic residues in the motif (Figure S4). E796 interacts with several residues adjacent to or in motif D that contribute to the motif dynamics, including K160, K780, K783, C799, and T801. In silico modeling shows that substitution E796D leads to a shorter side chain that might change the interaction patterns of this residue (Figure 7b), thus altering the dynamics of motif D and potentially changing the interaction of K798 with remdesivir, making its incorporation less favorable compared to ATP (Figure 7c).

## 4. Discussion

In this study, we characterize a SARS-CoV-2 variant exhibiting a decrease in remdesivir susceptibility and identify substitutions in the nsp12-RdRp and nsp14-ExoN that reduce drug susceptibility. Thus, long-term treatment with remdesivir led to the selection of substitution E796D in the drug target (nsp12) and substitution A225S in the nsp14-ExoN. Compared to previous remdesivir resistance in vitro studies [34,35,36,37,38,39], we designed experimental conditions to promote persistent SARS-CoV-2 infection with intermittent drug exposure, simulating persistent infection scenarios in immunosuppressed patients undergoing repeated remdesivir treatments [24,25,26,30,31,59]. Interestingly, our approach led to the identification of nsp12-E796D as an important substitution for resistance, and this same change was transiently detected in an immunocompromised COVID-19 patient after remdesivir treatment [59]. Additionally, nsp12-E796K was reported in another immunocompromised patient after repeated remdesivir treatments [30], highlighting the clinical relevance of substitutions at residue E796.

Previous in vitro studies have identified several remdesivir RAS in SARS-CoV-2 [34,35,36,37,38,39]. The relatively large number of different substitutions reported, likely influenced by differences in the experimental conditions across studies, emphasizes the evolutionary plasticity of SARS-CoV-2 in the development of remdesivir resistance. For example, the combination of nsp12-E796D and nsp14-A225S is unique to this study, whereas nsp12-E796D alone was observed in a remdesivir resistant virus reported by Schreiber et al. [39]. Similarly, Torii et al. reported another substitution at residue E796 (E796G) that correlated with remdesivir resistance [35]. Hence, multiple resistance studies are needed to fully cover the spectrum of SARS-CoV-2 remdesivir RAS, which is essential for the global surveillance of resistance.

Although RAS nsp12-E796D showed significant remdesivir resistance in replication assays, its effect on drug susceptibility in infectious assays was not evident. Indeed, the EC_50_ change observed for the polyclonal RDV escape virus was not fully recapitulated by any of the mutant viruses. This discrepancy was also observed in previous studies investigating nsp12-RAS V166L and E802D [34,37]. We hypothesize that additional viral factors beyond virus–drug interactions during replication, presumably mediated by drug-target substitutions, may contribute to remdesivir resistance in drug-escape studies performed in cell culture. In both our study and previous studies [34,37], the escape viruses acquired substitutions outside the drug target, beyond the RAS. These substitutions most likely reflect the evolution of SARS-CoV-2 in Vero E6 cells, leading to increased viral fitness [10]. A relationship between increased fitness and DAA resistance has been observed for other RNA viruses, including HCV [60,61] and the coronavirus murine hepatitis virus (MHV) [62]. In our study, the long-term resistance selection experiment might have simultaneously promoted the development of drug resistance and culture adaptation to Vero E6 cells. Increased fitness alone could not confer remdesivir resistance in SARS-CoV-2, as demonstrated in previous studies [10]. Consistent with this, the RDV untreated virus did not exhibit remdesivir resistance, despite having comparable fitness to the RDV escape virus.

Hence, we propose that the significant EC_50_ increase in the RDV escape virus in Vero E6 cells may have resulted from an interplay between RAS that reduce remdesivir susceptibility and other mutations that enhance viral fitness. In this context, the lack of fitness-enhancing mutations in the infectious mutants could explain why they do not reach the same EC_50_ value as the polyclonal RDV escape virus, despite clearly showing resistance in replication assays. Consistent with previous studies [21], the S759A+V792I control virus exhibited a substantial EC_50_-fold change in infectious assays, indicating that high-resistance mutations can drive significant EC_50_ changes independently of fitness. In contrast, RAS associated with moderate (no detectable-to-intermediate) resistance may benefit from the presence of fitness-enhancing mutations to amplify their resistance phenotype. To confirm this hypothesis, reverse genetics experiments will be needed to assess the contribution of non-RAS mutations in the RDV escape virus to both fitness and resistance. Moreover, specific fitness-enhancing mutations might differ according to cell types or in vivo. Thus, the relevance of this mechanism in vivo remains unclear. However, an accumulation of fitness-enhancing mutations was observed in the successively emerging SARS-CoV-2 variants [63], and remdesivir therapy has been linked to the emergence of SARS-CoV-2 variants with increased fitness in patients [64].

Similarly to nsp12-E796D, other in vitro-selected nsp12-RAS (V166A/L, N198S, V792I, C799F, and E802D [34,36,37]) have also been observed in immunocompromised COVID-19 patients treated with remdesivir [21,24,25,26,28,29,30,31,32,33]. However, the use of different methodologies to characterize these RAS between studies complicates their comparison. For example, three independent studies determined from 2.5- to 7.3-fold decrease in drug susceptibility for nsp12-E802D [32,35,37]. This study addresses these inconsistencies by providing a comprehensive classification of SARS-CoV-2 remdesivir RAS through head-to-head comparisons under standardized experimental conditions. For this, we chose a replicon system over an infectious system, as replicon systems allow for an evaluation of drug effectiveness without the interference of other viral factors, such as viral fitness, which we hypothesize influenced remdesivir resistance in our infectious system. Moreover, our replicon system has been used to study protease inhibitor resistance [20,65], and the isogenic infectious system has served to evaluate the efficacy of combination therapies against nirmatrelvir resistance [20], highlighting the utility of our platforms in antiviral resistance research.

A limitation of using replication fold-change values is the inability to directly infer EC_50_ values from a single treatment point. However, our results suggest that higher replication fold-changes correlate with increased EC_50_ values in infectious assays. Moreover, while absolute fold-change values differ between the two measures, the relative levels of resistance obtained here for the different RAS correlate well with the literature. Thus, the highest resistance levels in our replication assays were those of S759A and V792I combined, which also showed the highest EC_50_ increase in our study and all the previous reverse genetic analysis of remdesivir RAS performed in infectious systems [21,32,34,35,36,37], whether assessed in MHV [36] or in SARS-CoV-2 [21] infectious models. In contrast, RAS associated with moderate resistance in our replicon assay showed low and often overlapping EC_50_ values in prior studies [21,32,34,35,36,37]. In this context, our replicon assays proved more sensitive in quantifying the relative resistance levels conferred by RAS for which only small EC_50_ shifts were reported in the literature.

Moreover, the location of the different RAS in the nsp12 protein correlated with the levels of resistance obtained here, which may help predict the resistance associated with novel substitutions emerging after remdesivir treatment. In fact, there are nsp12-substitutions linked to remdesivir treatment failure in the clinic that have yet to be studied in vitro, such as A449V, R457C, and I536V [24,25,59]. Following our classification, none of them are expected to cause high remdesivir resistance, as A449V and R457C are located adjacent to, and I536V far from, the nsp12 active site. To date, most of the remdesivir RAS reported in COVID-19 patients and studied in vitro cause no detectable to intermediate levels of resistance [21,22,23,28,30,32,34,35,36,37], demonstrating that RAS causing moderate resistance are sufficient to promote treatment failure in the clinic. Moreover, as RAS outside the active site do not seem to negatively affect replication, as demonstrated with our assays, they might more easily emerge and propagate in COVID-19 patients. In that regard, we found that nsp12-E796D was genetically stable both in culture and in hamsters, which could explain its emergence in a remdesivir-treated COVID-19 patient [59]. Similarly, the intermediate RAS nsp12-V792I was found to be genetically stable and efficiently transmitted in hamsters [30]. On the contrary, substitutions located in the active site would most likely impair viral fitness, as seen for nsp12-S759A in this and previous studies [36]. Accordingly, nsp12-S759A has only been reported four times in the GISAID database, compared with the 292 viral sequences containing nsp12-E796D (CoVsurver at GISAID, accessed June 2025). An exception to this pattern is nsp12-RAS A376V, recently identified in a COVID-19 patient after remdesivir treatment. Despite being located outside the active site, A376V exhibited high levels of resistance and fitness impairment [23]. 

The location of RAS in the nsp12 protein has been correlated with various mechanisms of remdesivir resistance [66]. Our in silico modeling suggests that E796D could indirectly affect remdesivir incorporation by altering the dynamics of motif D in nsp12, influencing the interactions of the catalytic residue K798 (located in motif D) with remdesivir. Interestingly, a similar mechanism of resistance has been suggested for V166A and C799R/F [36]. V792I, which is also located in motif D, was shown to make ATP incorporation more favorable than remdesivir incorporation in biochemical assays [36]. Similarly, Sama et al. recently reported that a putative remdesivir RAS (nsp12-A777S) located near motif D increases the preference of ATP over remdesivir incorporation [38]. Thus, biochemical assays will be needed to confirm the predicted impact of E796D on remdesivir incorporation.

This is the first study associating a nsp14 substitution with decreased remdesivir susceptibility, as nsp14-A225S enhanced the resistance phenotype of nsp12-E796D. A225S is located close to the first zinc finger motif (ZF1) of nsp14-ExoN, in a position with no known role in RNA synthesis or proofreading. However, ZF1 was shown to be crucial for the structural stability and function of nsp14 in SARS-CoV [67]. Therefore, follow-up studies could explore whether nsp14-A225S contributes to remdesivir resistance by affecting the stability of nsp14.

In conclusion, we characterized substitutions correlated with decreased remdesivir susceptibility, one of which was reported in a remdesivir-treated patient. We also propose viral fitness as an important factor contributing to reduced susceptibility of SARS-CoV-2 to remdesivir in culture, which might also be relevant in vivo. Moreover, we provided a classification of SARS-CoV-2 remdesivir RAS, based on the correlation between drug susceptibility and location in the nsp12 protein, which can contribute to ongoing drug resistance surveillance efforts by acting as a reference when evaluating the resistance potential of novel substitutions. Altogether these data enhance our understanding of how SARS-CoV-2 evolves to escape the antiviral effect of the broad-spectrum antiviral remdesivir, which is a relevant compound for the treatment of other emerging viruses.

## Data Availability

The original contributions presented in this study are included in the article/Supplementary Materials. Further inquiries can be directed to the corresponding author.

## Supplementary Materials

The following supporting information can be downloaded at: https://www.mdpi.com/article/10.3390/v17081055/s1, Supporting Methods; Figure S1: Antiviral activity of remdesivir, GS-441524, obeldesivir, and molnupiravir; Figure S2: Competitive fitness of original and RDV escape viruses; Figure S3: Neutralizing curves of hamster plasma samples; Figure S4: Principal component analysis of nsp12–motif D in a nsp7-nsp8-nsp12 molecular dynamics simulation; Table S1: Genotypic characterization of remdesivir resistance selection experiment-derived viruses; Table S2: Genotypic characterization of mutant viruses; Table S3: Histopathological analysis of animal lungs.
