# Supplementary material for: SARS-CoV-2 Remdesivir Exposure Leads to Different Evolutionary Pathways That Converge in Moderate Levels of Drug Resistance
Source: Viruses. 2025 Jul 29;17(8):1055. doi: 10.3390/v17081055 (PMC12390670; doi:10.3390/v17081055)
Supplement: Supplementary file 1 [file viruses-17-01055-s001.zip › viruses-3738797-supplementary.pdf]

Supplementary Material Fernandez-Antunez et al.

Supporting Methods.

Remdesivir Resistance Selection Experiment: For the remdesivir resistance selection experiment, cells infected with the original virus were treated with increasing drug concentrations for three consecutive passages (passages 1, 2, and 3). In passage 1, cells were infected with the original virus (MOI 0.1, calculated using infectivity titers) and after 33 days of infection, cells were treated with 2 μM of remdesivir until day 48 post-infection, when treatment was interrupted to avoid viral clearance (as no CPE was observed in the culture). The culture was terminated 80 days post-infection, when massive cell death was observed, presumably due to viral propagation. In passage 2, a new infection was performed with 100 μL of culture supernatant (MOI 0.3, calculated using infectivity titers) harvested from passage 1 on day 80 post-infection. Treatment with 4 μM of remdesivir was initiated 24h post-infection and further increased to 8 μM at day 11 post-infection. High levels of CPE were observed throughout passage 2. In passage 3, cells were infected with 100 μL of culture supernatant (MOI 0.003, calculated using infectivity titers) harvested at passage 2, day 14 post-infection. Treatment with 10 μM of remdesivir was initiated 1h post-infection and maintained until day 4 post-infection, when treatment was interrupted to avoid viral clearance. Treatment was resumed on day 11 post-infection with 40 μM of remdesivir and interrupted again at day 13. The culture was terminated at day 18 post-infection, when massive CPE was observed. A non-treated infected control culture was maintained for the three consecutive passages, for which infections were performed in similar conditions as for the treated virus (MOI 0.1 for passage 1, MOI 0.2 for passage 2, and MOI 0.5 for passage 3, calculated using infectivity titers). In passages 1 and 2, culture was maintained until 80 and 14 days post-infection, respectively. In passage 3, culture was only maintained until day 6 post-infection due to massive CPE.

Reverse Genetics Mutagenesis: An In-Fusion PCR-based technique was used for the introduction of mutations into the original replicon, a SARS-CoV-2 subgenomic reporter replicon system based on the SARS-CoV-2/human/Denmark/DK-AHH1/2020 isolate sequence [42]. In brief, overlapping fragments of the subgenomic replicon system template with the desired mutations were generated by PCRs using the Q5® Hot Start High-Fidelity 2X Master Mix, according to the manufacturer's instructions. PCR products were purified with the Zymoclean™ Gel DNA Recovery Kit with Zymo-Spin™ IC-XL columns from the ZR BAC DNA Miniprep Kit (ZymoResearch) and further used for the In-Fusion reaction using the In-Fusion® HD Cloning Kit (Takara bio, Kusatsu, Japan), following the manufacturer's instructions.

For the introduction of mutations into the original clone, a full-length SARS-CoV-2 infectious system based on the SARS-CoV-2/human/Denmark/DK-AHH1/2020 isolate sequence [42], a megaprimer PCR-based technique was used instead. In brief, megaprimers (≈2000 bp) were generated by PCRs using the Q5® Hot Start High-Fidelity 2X Master Mix (New England Biolabs) according to the manufacturer's instructions. PCR products were then purified with the Zymo DNA clean & concentrator-25 kit (ZymoResearch) and further used for the site-directed mutagenesis of the template, using the Q5® Hot Start High-Fidelity 2X Master Mix and the following PCR conditions: 98 °C for 30 s, 20 cycles of 98 °C for 10 s, 48 °C for 1 min, and 72 °C for 35 min, followed by a final extension at 72 °C for 35 min. The final PCR product was digested with FastDigest DpnI (Thermo Fisher) before proceeding to downstream cloning.

RNA Transfections: RNA transcripts of replicon or full-length clones were transfected into cells using lipofectamine 2000 (Thermo Fisher). In brief, RNA diluted in Opti-MEM (Thermo Fisher) was mixed at a 1:1 ratio with lipofectamine diluted 1:50 in Opti-MEM. After 20 min of incubation at RT, 500 µL of the RNA/lipofectamine mix were added to cells containing Opti-MEM. For the transfection of replicon clones, cells were pre-incubated with the specified remdesivir concentrations from 16h prior transfection. Replacement of Opti-MEM with regular media supplemented with the specified remdesivir concentrations was performed 1h post-transfection. For the transfection of full-length clones, Opti-MEM was replaced by regular media 3h post-transfection.

Processing of Lung Tissue for Histological Evaluation: To perform a histopathological analysis of lung tissue, the right lung from each animal was immersion fixed in 10% buffered formalin and post-fixed for 24–48h upon euthanasia, and then stored in 70% ethanol until trimming, tissue processing, and paraffin embedding. Tissues were then sectioned at 5 µm onto slides, deparaffinized, and stained with hematoxylin and eosin for histological evaluation.

Next-Generation Sequencing Analysis: The nearly full-length SARS-CoV-2 genome was amplified from viral RNA in five overlapping amplicons through RT-PCR using the Maxima H Minus Reverse Transcriptase (Thermo fisher) and Q5® Hot Start High-Fidelity 2X Master Mix. The five amplicons were then purified with the Zymo DNA clean & concentrator-25 kit and pooled at a 1:1 ratio. Library preparation was performed using the NEBNext Ultra II FS DNA Library Prep Kit (New England Biolabs), with a fragmentation size of 500 bp. Samples were size-selected using Ampure XP beads (Beckman Coulter, Brea, CA, US) and multiplexed with index primers (New England Biolabs). Library preparations (4 nM) were pooled for NGS, which was conducted in-house using Illumina Miseq (Illumina), using the v2 500 cycle kit with the 250 bp pair-end setting. For the NGS analysis, the PCR primer sequences at the 5′ end of all reads were trimmed by Cutadapt to remove the bias of the 5′-overlapping amplicons. Reads were then mapped to the SARS-CoV-2 reference sequence by BWA MEM, and processed by SAMtools. Low-frequency single-nucleotide polymorphisms (SNPs) were identified using Lo-Freq, translated by SnpEff, and detected by VCFTools with a 0.5% cutoff. Plasmid sequences were also analyzed by NGS and confirmed with the Geneious Prime software.

Supporting Results.

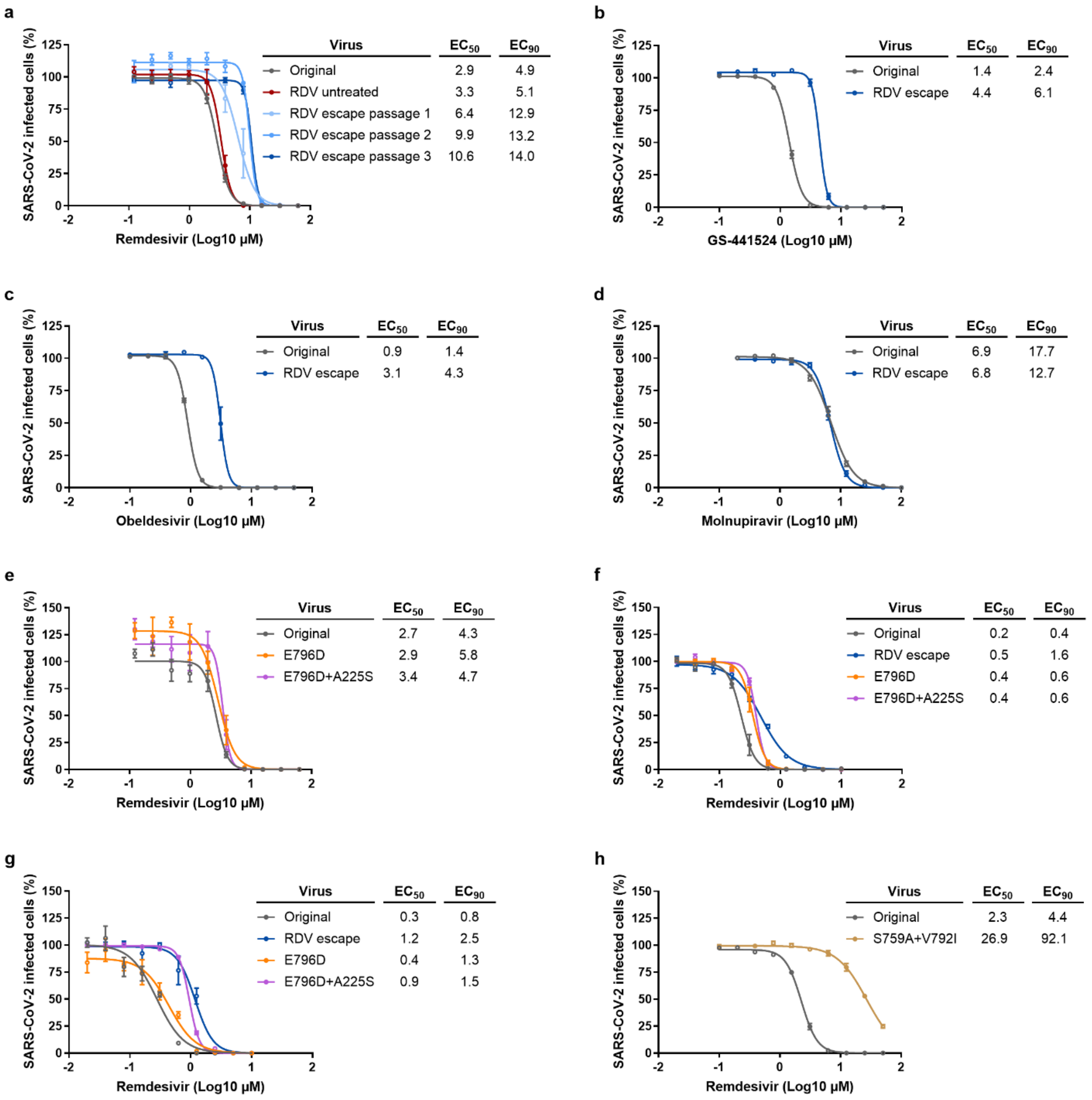

Figure S1. Antiviral activity of remdesivir, GS-441524, obeldesivir, and molnupiravir. Drug concentration-response assays were performed in Vero E6 cells for (a,e,h) remdesivir, (b) GS-441524, (c) obeldesivir, and (d) molnupiravir. Remdesivir concentration–response assays were also performed in human cell-lines, (f) Calu-3, and (g) A549-hACE2. Graphs show the non-linear regression curves of SARS-CoV-2-infected cells normalized to nontreated controls (Y-axis, in percentage) of different viruses across various drug concentrations (X-axis, $\log_{10}$ µM). Symbols represent the mean of four replicates, with error bars indicating the standard error of the mean. The EC_{50} and EC_{90} values for the different viruses tested are also indicated. The S759A+V792I positive control mutant maintained the introduced mutations, with no further acquisition of any substitution.

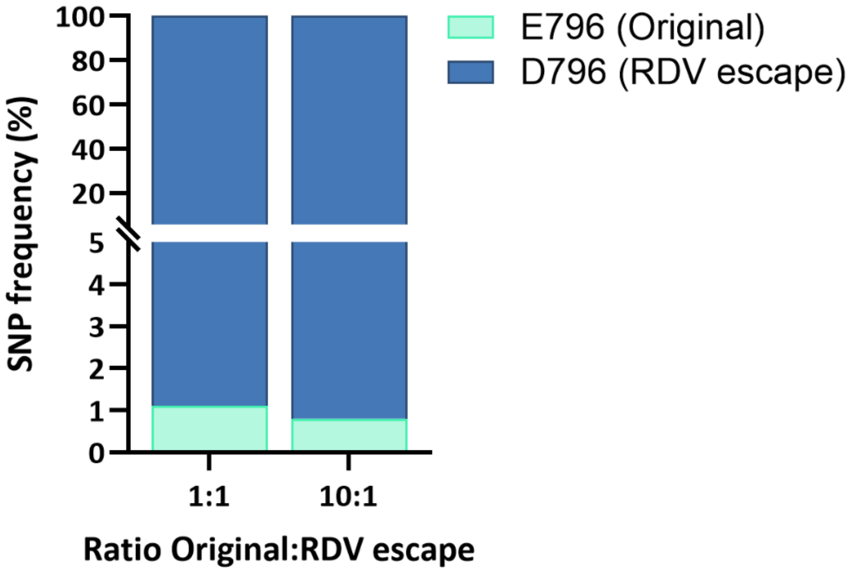

Figure S2. Competitive fitness of original and RDV escape viruses. Vero E6 cells were co-infected with mixtures of original and RDV escape viruses at either 1:1 (MOI 10^{-4}:10^{-4}) or 10:1 (MOI 10^{-4}:10^{-5}) input ratios, respectively. The graph shows the frequency (%) of virus-specific SNP at position nsp12-796 (Y-axis), measured in supernatants harvested 72h after co-infection at the indicated input ratios (X-axis).

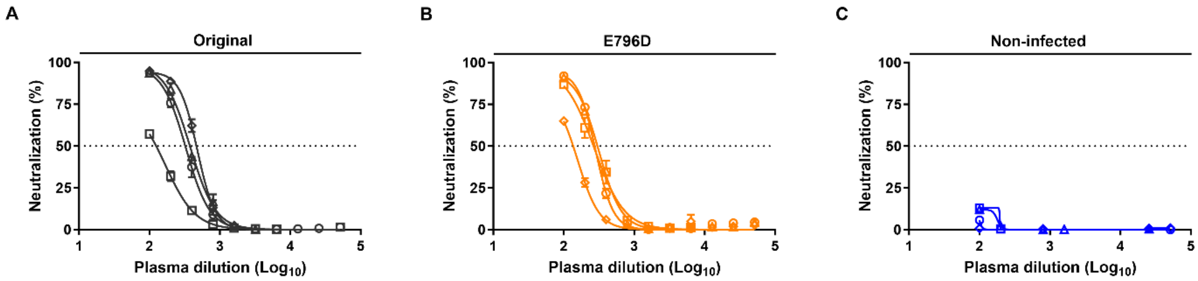

Figure S3. Neutralizing curves of hamster plasma samples. Graphs show the neutralization of the original virus by animal plasma samples collected upon euthanasia as non-linear regression curves of viral inhibition normalized to non-treated controls (Y-axis, in percentage) for the different plasma dilutions (X-axis, $\log_{10}$) from (a) animals infected with the original virus, (b) animals infected with the E796D mutant virus, and (c) non-infected animals. Each individual animal is represented by a different symbol, and symbols represent the mean of quadruplicates, with error bars representing standard errors of the mean. The dotted line highlights the 50% neutralization level.

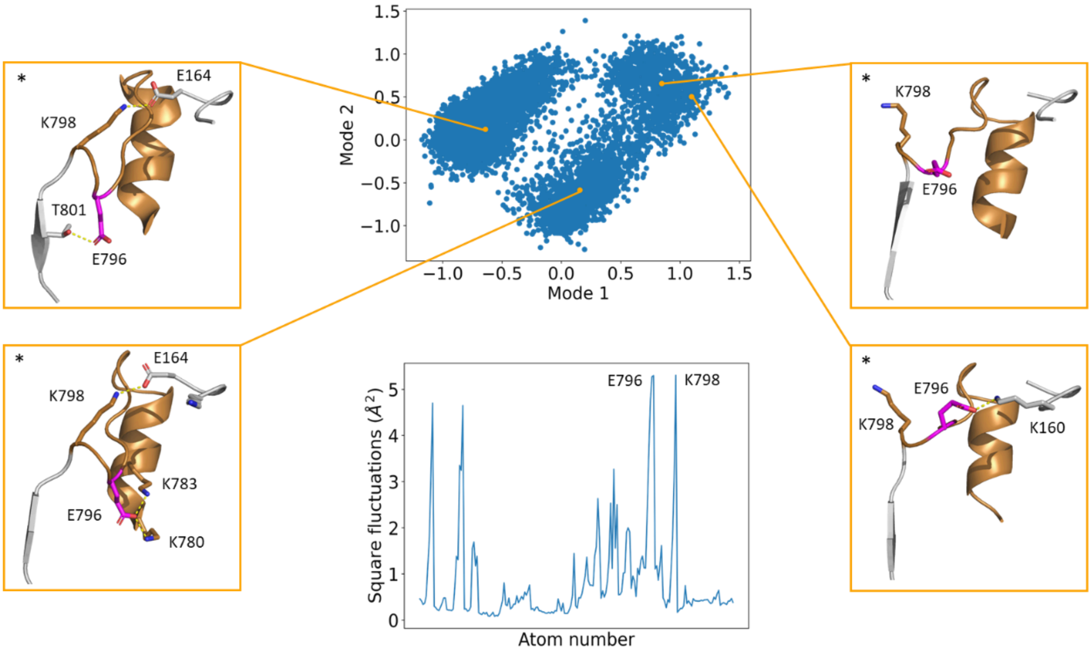

Figure S4. Principal component analysis of nsp12–motif D in a nsp7-nsp8-nsp12 molecular dynamics simulation. The upper plot shows the projections of normal mode 1 and normal mode 2 from the principal component analysis. Example structures from different timepoints in the molecular dynamics simulation are shown in orange rectangles to illustrate the conformational flexibility of nsp12–motif D (brown) and the interactions of E796 (magenta) and K798 with other residues. Dashed yellow lines indicate interactions between residues. * indicates the location of the nsp12 active site. The lower plot shows the square fluctuations (Y-axis) calculated in the principal component analysis of nsp12–motif D against atom number (X-axis). Residues E796 and K798, corresponding to the two major peaks, are annotated.

Table S1. Genotypic characterization of remdesivir resistance selection experiment-derived viruses.

SARS-CoV-2 genomic region	nsp2		nsp3			nsp4	nsp6	nsp7	nsp12				nsp14		nsp16
Nt number	1059	1926	3884	7160	7787	9546	11522	12015	14408	14749	15814	15828	18712	18928	20999
Nt Whuhan-Hu-1	**C**	**C**	**C**	**G**	**G**	**T**	**T**	**T**	**C**	**T**	**G**	**A**	**G**	**C**	**A**
Nt change	T	T	T	C	T	C	G	G	T	G	C	C	T	T	G
RDV untreated	99.8	25.5	62.8	38.3	•	•	7.9	5.1	99.8	•	•	•	•	29.8	•
RDV escape passage 1 day 64	99.6	•	•	•	32.7	•	55.7	•	99.4	•	36.9	11.7	•	•	•
RDV escape passage 1	99.7	•	•	•	18.3	•	76.8	•	99.8	•	39.3	9.3	•	•	•
RDV escape passage 2 day 14	99.9	•	•	•	•	83.1	•	6.1	99.9	83.3	•	99.9	99.8	•	•
RDV escape passage 2	99.7	•	•	•	•	94.6	1.3	4.5	99.8	94.9	•	99.4	99.3	•	•
RDV escape passage 3 day 18	99.8	•	•	•	•	1.9	2.3	97.5	99.8	2.2	•	99.6	99.8	•	96.1
RDV escape passage 3	99.5	•	•	•	•	1.9	3.7	97.0	99.6	2.0	•	99.3	99.2	•	94.4
Specific aa protein number	85	374	389	1481	1690	331	184	58	323	437	792	796	225	297	114
Aa change from Whuhan-Hu-1	T-I	T-I	P-S	D-H	A-S	L-S	F-V	V-G	P-L	L-V	V-L	E-D	A-S	P-S	D-G

SARS-CoV-2 genomic region	S																ORF3a	E			ORF7a	ORF8	N
Nt number	21575	21764-21770	21849	22016	22036	22117	22199	22661	23014	23403	23615	23922	23997	24000	24374	24538	25563	26335	26380	26423	27619	27987	28899
Nt Whuhan-Hu-1	**C**	**ATACATG**	**A**	**T**	**A**	**T**	**G**	**G**	**A**	**A**	**C**	**A**	**C**	**G**	**C**	**A**	**G**	**C**	**A**	**C**	**C**	**G**	**G**
Nt change	T	A	C	A	C	G	A	T	T	G	A	C	T	T	T	C	T	A	G	A	T	A	T
RDV untreated	•	67.5	99.8	•	•	99.6	•	68.7	71.7	99.8	99.4	•	99.6	•	37.4	99.6	99.9	•	•	99.5	•	99.6	99.4
RDV escape passage 1 day 64	46.2	•	86.7	11.2	•	86.4	35.7	•	•	99.5	99.2	•	52.9	18.1	•	•	99.5	13.5	44.6	39.3	•	•	98.7
RDV escape passage 1	46.9	•	90.9	13.6	•	86.6	40.2	•	•	99.5	99.4	•	53.9	36.3	•	•	99.7	14.2	44.2	40.4	•	•	99.6
RDV escape passage 2 day 14	•	•	•	99.8	1.0	•	•	•	99.9	99.8	99.7	82.9	•	99.9	•	•	99.9	99.9	•	•	16.9	•	99.8
RDV escape passage 2	•	•	•	99.5	1.9	•	•	•	99.6	99.6	99.4	94.7	•	99.6	•	•	99.8	99.6	•	•	4.5	•	99.7
RDV escape passage 3 day 18	0.4	•	•	99.7	96.1	•	•	•	99.8	99.7	99.7	1.9	•	99.8	•	•	99.9	99.8	•	•	41.9	•	99.7
RDV escape passage 3	0.6	•	•	99.6	95.2	•	•	•	99.7	99.4	99.2	1.9	•	99.3	•	•	99.6	99.5	•	•	49.2	•	99.4
Specific aa protein number	5	69-70	96	152	158	185	213	367	484	614	685	787	812	813	938	992	57	31	46	60	76	32	209
Aa change from Whuhan-Hu-1	L-F	Δ2	E-A	W-R	R-S	N-K	V-M	V-F	E-D	D-G	R-S	Q-P	P-L	S-I	L-F	Q-H	Q-H	L-I	I-V	S-Y	Q-X	V-I	R-I

The table shows NGS results as the frequency (in percentage) of nucleotide changes leading to amino acid substitutions present through the near-full-length genome sequence of different viruses (left column) compared to the Wuhan-Hu-1 isolate (genbank NC045512.2) (top). Viruses directly derived from each passage of the resistance selection experiment are indicated in blue, whereas viruses obtained after a drug-free passage are indicated in black. No change compared to Wuhan-Hu-1 is indicated as "●". Substitutions present in the RDV untreated and RDV escape passages 1, 2, and 3 viruses from the original virus are indicated by grey shading. The SARS-CoV-2 genomic regions, nucleotide (nt) number, nucleotide change, specific amino acid (aa) protein number, and amino acid changes from Wuhan-Hu-1 are indicated. All substitutions with frequency ≥20% are shown, although the frequency <20% of substitutions present in one of the viruses at ≥20% is also indicated in other viruses. Frequency values are rounded to the nearest decimal.

Table S2. Genotypic characterization of mutant viruses.

SARS-CoV-2 genomic region	nsp2	nsp12		nsp14	S			ORF3a	N
Nt number	1059	14408	15828	18712	22487	23403	23606	25563	28899
Nt Whuhan-Hu-1	**C**	**C**	**A**	**G**	**G**	**A**	**C**	**G**	**G**
Nt change	T	T	C	T	A	G	T	T	T
E796D	99.5	99.6	99.3	•	99.1	99.5	31.5	99.6	99.4
E796D+A225S	99.7	99.8	99.7	99.5	99.5	99.7	1.2	99.8	99.2
Specific aa protein number	85	323	796	225	309	614	682	57	209
Aa change from Whuhan-Hu-1	T-I	P-L	E-D	A-S	E-K	D-G	R-W	Q-H	R-I

The table shows the frequency (in percentage, analyzed by NGS) of nucleotide changes leading to amino acid substitutions present in the near full-length genome sequence of mutant viruses (left column), compared to the Wuhan-Hu-1 isolate (genbank NC045512.2) (top). No change compared to Wuhan-Hu-1 is indicated as "●". Substitutions present in the different viruses from the original clone are indicated by grey shading. Engineered substitutions are indicated by orange shading. The SARS-CoV-2 genomic regions, nucleotide (nt) number, nucleotide change, specific amino acid (aa) protein number, and amino acid changes from Wuhan-Hu-1 are indicated. All substitutions with frequency ≥5% are shown, although frequencies of <5% of substitutions present in one of the viruses at ≥5% are also indicated in other viruses. Frequency values are rounded to the nearest decimal.

Table S3. Histopathological analysis of animal lungs.

Group	Original				E796D				Non-infected			
**Animal**	**1**	**2**	**3**	**4**	**1**	**2**	**3**	**4**	**1**	**2**	**3**	**4**
**Bronchioli**												
Epithelial necrosis (− /+)[1]	+ terminal	+ terminal	+ terminal	+ terminal	+	+	+	+	−	−	−	−
Inflammation (− /+/++)[2]	+	+	+	++	+	+	−	+	−	−	−	−
- macrophages (− /+/++)[2]	+	+	+	+	+	+	−	+	−	−	−	−
- neutrophil granulocytes (− /+/++)[2]	+	+	+	+	+	+	−	+	−	−	−	−
Epithelial hyperplasia (− /+)[1]	+	+	−	+	+	+	+	+	−	−	−	−
Edema in mucosa and submucosa (− /+)[1]	−	−	−	−	−	−	−	−	−	−	−	−
**Alveolar tissue**												
Inflammation (− /+/++)[2]	++	++	++	++	+	++	+	++	−	−	−	−
- macrophages (− /+/++)[2]	+	+	+	+	+	+	+	+	−	−	−	−
- neutrophil granulocytes (− /+/++)[2]	+	+	+	+	+	+	+	+	−	−	−	−
Necrosis (− /+)[1]	+ focal	+ focal	+	+	−	+	+	+	−	−	−	−
Edema (− /+)[1]	−	+ perivascular	+ perivascular	+ perivascular	+	+ perivascular	−	−	−	−	−	−
Hyperemia (− /+)[1]	−	−	−	−	−	−	−	−	−	−	−	−
Type II pneumocyte hyperplasia (− /+)[1]	+	+	+	+	+	+	−	+	−	−	−	−
Syncytial cell formation (− /+)[1]	+	+	+	−	−	+	−	+	−	−	−	−
Adherent mononuclear cells to endothelium in vessels (− /+)[1]	+	−	+	+	+	+	−	+	−	−	−	−

The table shows the scoring of histopathological parameters (left column) in the animals of the different experimental groups (top). [1] -, absent / +, present. For some categories, the extent or location is detailed. [2]-, absent / +, few / ++, numerous. Of note, all animals infected with the original virus, and animals 1, 2, and 4 infected with the E796D mutant virus, showed signs of bronchiolitis.
